# Assessment of angiogenic factor, vascular endothelial growth factor, serum and urine level changes in superficial bladder tumor immunotherapy by intravesical Bacillus Calmette-Guerin

**DOI:** 10.4103/0974-7796.68855

**Published:** 2010

**Authors:** Behzad Feizzadeh Kerigh, Abdolazim Bahrami, Ali Shamsa, Mehran Abolbashari

**Affiliations:** Endoscopic and Minimally Invasive Surgery Research Center, Mashad, Iran; 1 Mashad University of Medical Sciences, Ghaem Hospital, Mashad, Iran

**Keywords:** Angiogenic factor, intravesical Bacillus Calmette-Guerin, superficial bladder tumors, vascular endothelial growth factor

## Abstract

**Background and Aim:**

Bladder tumor is one of the most common genitourinary tumors. Management of non-muscle invasive (NMI) bladder tumors is primarily by transurethral resection (TURBT) followed by intravesical immunotherapy or chemotherapy. Bacillus Calmette-Guerin (BCG) is the most effective adjuvant therapy in NMI bladder tumor. Since angiogenesis is an essential factor in solid tumor progression and vascular endothelial growth factor (VEGF) is an important factor in angiogenesis, the aim of this study is the assessment of angiogenic factor, VEGF, serum and urine level changes in superficial bladder tumor immunotherapy by intravesical BCG.

**Materials and Methods:**

A total of 23 patients with bladder transitional cell carcinoma (TCC) in stage Ta/T1 or carcinoma *insitu* (CIS), low or high grade, which passed a 2-4 week period from TURBT participated in this study. Blood and urine samples were obtained at first and sixth sessions before instillation of BCG. Enzyme-linked immunosorbent assay (ELISA) method was used to obtain VEGF level in samples.

**Results:**

Urine and serum VEGF levels did not change significantly before and after BCG therapy. Changes in VEGF level were significantly different neither in low grade against high grade tumors nor in stage T1 against stage Ta tumors. A significant difference in VEGF level was seen between low grade and high grade tumors in serum after BCG therapy (*P*=0.007); but not in urine samples.

**Conclusion:**

Although intravesical BCG possesses anti-angiogenic activity, it seems that it exerts its effect through pathways other than VEGF, especially in low grade tumors.

## INTRODUCTION

Bladder tumor is one of the most common genitourinary tumors. It is the fourth common cancer in men after prostate, lung, and colorectal cancers; and accounts for 6.8% of cancer cases.[1] Mortality due to bladder cancer is relatively high. Also, 13,180 cases of deaths were reported due to bladder cancer in USA in the year 2005.[1]

More than 90% of bladder cancers are transitional cell carcinomas in which 70% are non-muscle invasive (NMI) including tumors in stage Ta (70%), T1 (20%) and carcinoma *in situ* (10%).[2] Management of NMI bladder tumors is primarily by transurethral resection (TURBT) followed by intravesical immunotherapy or chemotherapy.[3–5]

Bacillus Calmette-Guerin (BCG) is the most effective adjuvant therapy that has succeeded in reduction of recurrence rate and progression of bladder transitional cell carcinoma (TCC).[6] Despite the widespread use of BCG in TCC of the bladder, its mechanism is not well recognized. On the other hand, many studies have shown the role of angiogenesis in tumor progression and spread.[7–9] Many factors are included in angiogenesis among which vascular endothelial growth factor (VEGF) seems to be one of the most important components.[10,11] VEGF is a principal regulator of angiogenesis, which induces proliferation and differentiation of endothelial cells. VEGF had been used as a diagnostic indicator in bladder tumor.[12,13]

Overexpression of VEGF has been shown in high stage bladder tumor. VEGF level also has an impact on prognosis and mortality rate of bladder cancer. Drugs that aimed on VEGF and their pathways have shown encouraging results for tumor therapy in different studies.[14–19]

The aim of this study is the assessment of angiogenic factor, VEGF, serum and urine level changes in superficial bladder tumor immunotherapy by intravesical BCG. To our knowledge, such an issue has not been investigated yet.

## MATERIALS AND METHODS

This is a prospective and non-randomized study. Twenty-three patients with NMI TCC bladder tumor, who were referred to our center for BCG therapy from April 2006 to September 2008, were enrolled in this study based on the following criteria.

Inclusion criteria were bladder TCC in stage Ta/T1 or carcinoma insitu (CIS), low or high grade, which passed a 2–4 week period from TURBT. The exclusion criteria were tuberculosis history, clinical active urinary tract infection, total urinary incontinence, pregnancy, immunosuppressed patients, gross hematuria, hepatic disease, stage T2 or higher. Indications to discontinue BCG were urethral injury during catheterization, tuberculosis-induced sepsis, fever with temperature more than 38.5°C for 48 hours during BCG therapy or any occurrence of fever with temperature >40°C.

All the patients were informed about the study and informed consent was taken from all of them. This study was approved by ethical committee of Mashhad University of Medical Sciences.

Besides BCG therapy that was a therapeutic part in the study; other interventions were blood sampling (2 ml) from vein and urine sampling through voided urine.

Blood and urine samples were obtained at first session of BCG therapy before instillation of BCG; also, sixth sessions of BCG therapy were performed. Pasteur institute BCG vials, which contained 120 mg BCG, were used. At the sixth session, before instillation of BCG, blood and urine samples were obtained. Blood samples were centrifuged to separate serum. Then these samples, as well as urine samples, were refrigerated at –20°C.

To obtain VEGF level in samples, enzyme-linked immunosorbent assay (ELISA) method was used. Samples were warmed at room temperature. Serum samples were directly treated and urine samples were examined by 1/2 dilution. Human VEGF ELISA development kit (Duoset from R and D system, Minneapolis, MN, USA) was used. Samples were placed adjacent to the antibody, and after being washed for several times, they were read using a micro plate reader.

BCG vial was diluted with 50 ml normal saline for instillation of BCG, and then it was infused through a 8-Fr. urethral catheter. Before instillation, the patient was asked to void urine to empty the bladder completely; after instillation of BCG, the patient was instructed to retain urine for at least 2 hours.

Data were analyzed by SPSS software (version 11). The t test and non-parametric tests were used to evaluate data.

## RESULTS

A total of 23 patients with NMI bladder TCC, who were referred to our center for BCG therapy, from April 2006 to September 2008, and who fulfilled the including criteria were assessed.

Among them were 21 (91.3%) males and 2 (8.7%) females. The patients were 25–80 years old (62±13). Sixteen patients presented with gross hematuria and then diagnosed to have TCC.

One patient’s chief complaint was dysuria. The remaining six patients were diagnosed incidentally by microscopic hematuria in urine analysis or through an ultrasonography of bladder. There was no RBC in urine analysis of five patients.

Symptoms of more advanced disease were present in one patient who complained of flank pain. But the pathologic results showed NMI disease in this case. Irritative symptoms were present in 13 (65%) patients.

One-third of the patients had history of smoking. They had smoked for 20–50 years and with an average of 1.25 packs per day.

Majority of the patients were in stage T1 (61%) and others were in stage Ta. On grading, there were 4 patients with high grade tumor and 19 patients with low grade tumor. All high grade tumors were of stage T1 on pathology and all were males.

VEGF level was assessed using ELISA method. Serum VEGF levels before and after BCG therapy were 31–2000 pg/ml (488±498) and 105–1860 pg/ml (516±502), respectively. Urine VEGF levels before and after BCG therapy were 0–2000 pg/ml (153±424) and 0–2000 pg/ml (132±413), respectively.

There was no significant difference in VEGF levels due to BCG therapy in serum and urine (*P*=0.86 for both pairs) [Table 1].

**Table 1:** Urine and serum VEGF before and after BCG therapy

	Total (pg/ml)	*P* value
Urine VEGF before BCG therapy	153±424	0.86
Urine VEGF after BCG therapy	132±413	
Serum VEGF before BCG therapy	488±498	0.86
Serum VEGF after BCG therapy	516±502	

Patients in low grade and high grade groups were compared according to VEGF level [Table 2]. In both the groups, urine VEGF was reduced after BCG therapy; however, this reduction was more in high grade group. Serum VEGF level was reduced in high grade group but not statistically significant difference was observed. Serum VEGF level change in low grade group was not significant.

**Table 2:** Urine and serum VEGF according to tumor grade

	Low grade (pg/ml)	High grade (pg/ml)	*P* value	Total (pg/ml)
Urine VEGF before BCG therapy	149±451	172±308	0.90	153±424
Urine VEGF after BCG therapy	136±452	118±149	0.89	132±413
Serum VEGF before BCG therapy	526±523	334±102	0.17	488±498
Serum VEGF after BCG therapy	571±524	189±53	0.007	516±502

Groups were stratified according to stage [Table 3]. In T1 group, VEGF level reduced in serum, but increased in urine after BCG therapy. In Ta group, VEGF level did not differ after BCG therapy.

**Table 3:** Urine and serum VEGF according to tumor stage

	Ta (pg/ml)	T1 (pg/ml)	*P* value	Total (pg/ml)
Urine VEGF before BCG therapy	30±28	122±193	0.17	153±424
Urine VEGF after BCG therapy	34±34	265±617	0.27	132±413
Serum VEGF before BCG therapy	747±720	500±405	0.47	488±498
Serum VEGF after BCG therapy	793±734	399±318	0.26	516±502

VEGF levels in urine and serum before and after BCG therapy were compared according to the grade of tumor. A significant difference in VEGF levels was seen between low grade and high grade tumors in serum after BCG therapy (*P*=0.007).

## DISCUSSION

Intravesical BCG therapy is an effective treatment for NMI bladder TCC.[3] BCG mechanism is not defined well; but many theories have been proposed.[3,20] One of the important mechanisms of BCG in NMI TCC is inhibition of neovascularization, which is mediated through several factors.[21,22] Interferon-gamma (IFN-γ), interferon-inducible protein-10 (IP-10), and tumor necrosis factor-alpha (TNF-α) are among the factors that are increased after BCG therapy. These factors exert angiogenic effects.[22]

VEGF is one of the principal factors in angiogenesis.[10] VEGF is increased in bladder tumor and this increase is influenced by tumor stage and grade.[23,24] VEGF also has an effect on prognosis and recurrence of bladder tumor.[25] This study was designed to assess the changes of VEGF level in serum and urine after BCG therapy.

Age distribution was comparable with TCC distribution worldwide. Patients’ average age was 62.6 years that was comparable with that of Jemal *et al*.’s study.[1] TCC occurred more often in males. Gross hematuria was the presenting symptom in majority of the patients. Only 30% of patients were free of gross hematuria at presentation. This shows the importance of gross hematuria and necessity of diagnostic work-up in aged patients with hematuria. Smoking is one the risk factors for bladder tumor, attributing to the condition in 30% of patients. In this study, 30% of patients had the history of smoking.

VEGF measurement was performed by ELISA method. This method was frequency used in several studies.[21,22] In this study, although VEGF level was reduced in urine after BCG therapy, but this change was not statistically significant. Pavlovich *et al*. showed that VEGF increases in urine after BCG therapy. This finding is in contrast with our findings. This difference may be attributed to small sample size in Pavlovich’s study.[22] Morgan *et al*. demonstrated that VEGF in bladder tissue does not change significantly after BCG therapy. However, in 24 patients who showed no recurrence, tissue VEGF was not identifiable. Serum VEGF changed slightly after BCG therapy; however, this change was not significant.[21]

Urine VEGF level was lower in low grade tumors than in high grade tumors and the reduction was lesser in low grade patients after BCG therapy. But Beecken *et al*. found a higher VEGF level in low grade tumors.[23] This may partially be due to incorporation of muscle invasive tumors in their study. The paucity of high grade tumors in our study may justify this difference. Serum VEGF was higher in low grade TCC before BCG therapy than in high grade TCC. This finding is in accordance with Beecken’s study. Serum VEGF level after BCG therapy was related to tumor grade. This has not been mentioned in previous studies.[21,22] As high grade is related to recurrence and progression of tumor, we can assume that lower serum VEGF after BCG therapy predicts recurrence and progression of NMI bladder tumor. But Santos *et al*. found a reverse relationship between tumor recurrence and serum VEGF level.[25]

This study revealed no relationship between tumor stage and VEGF level but some studies have reported such a relationship.[26]

This study investigated the relationship between VEGF level in urine and serum before and after BCG therapy. In two previous studies, such a relationship was not found.[21,22] This study differs from previous studies in that urine and serum VEGF were assessed simultaneously in this study. This study, as other earlier studies, suggests that BCG mechanism may be through pathways other than VEGF pathways. But this theory awaits other studies to be confirmed.

If BCG mechanism for angiogenesis inhibition does not include VEGF as confirmed in this study, VEGF can be a therapeutic target in NMI bladder TCC.

This hypothesis has been examined in different studies. Rocchetti *et al*. showed that anti-VEGF drugs have a potential benefit in bladder tumors that express VEGF.[27] However, anti-VEGF drugs in bladder tumor therapy are considered investigational yet.

Although intravesical BCG is effective in NMI bladder tumor, and reduces recurrence and progression,[3] its complications are problematic. Different studies have tried to reduce BCG dose to decrease its complications, while maintaining its efficacy.[28,29] In spite of its complications, intravescial BCG has remained as the first line therapy in high grade tumors.

Intravesical anti-VEGF drugs may open a new window in the treatment of NMI TCC. It can be administered in conjunction with intravesical BCG to reduce its dosage and may substitute BCG in specific groups.

## CONCLUSION

Although intravesical BCG possesses anti-angiogenic activity, it seems that it exerts some effects through pathways other than VEGF (especially in low grade tumors). Hence, intravesical anti-VEGF drugs may have a potential role in adjuvant therapy of NMI TCC, which might reduce BCG complications.
